# Towards a general approach for tailoring the hydrophobic binding site of phenylalanine ammonia-lyases

**DOI:** 10.1038/s41598-022-14585-0

**Published:** 2022-06-23

**Authors:** Souad Diana Tork, Mădălina Elena Moisă, Lilla Cserepes, Alina Filip, Levente Csaba Nagy, Florin Dan Irimie, László Csaba Bencze

**Affiliations:** Enzymology and Applied Biocatalysis Research Center, Faculty of Chemistry and Chemical Engineering, Babeș-Bolyai University, Arany János Street 11, 400028 Cluj-Napoca, Romania

**Keywords:** Biocatalysis, Enzymes, Protein design

## Abstract

Unnatural substituted amino acids play an important role as chiral building blocks, especially for pharmaceutical industry, where the synthesis of chiral biologically active molecules still represents an open challenge. Recently, modification of the hydrophobic binding pocket of phenylalanine ammonia-lyase from *Petroselinum crispum* (*Pc*PAL) resulted in specifically tailored *Pc*PAL variants, contributing to a rational design template for PAL-activity enhancements towards the differently substituted substrate analogues. Within this study we tested the general applicability of this rational design model in case of PALs, of different sources, such as from *Arabidopsis thaliana* (*At*PAL) and *Rhodosporidium toruloides* (*Rt*PAL). With some exceptions, the results support that the positions of substrate specificity modulating residues are conserved among PALs, thus the mutation with beneficial effect for PAL-activity enhancement can be predicted using the established rational design model. Accordingly, the study supports that tailoring PALs of different origins and different substrate scope, can be performed through a general method. Moreover, the fact that *At*PAL variants I461V, L133A and L257V, all outperformed in terms of catalytic efficiency the corresponding, previously reported, highly efficient *Pc*PAL variants, of identical catalytic site, suggests that not only catalytic site differences influence the PAL-activity, thus for the selection of the optimal PAL-biocatalysts for a targeted process, screening of PALs from different origins, should be included.

## Introduction

**Figure 1:**
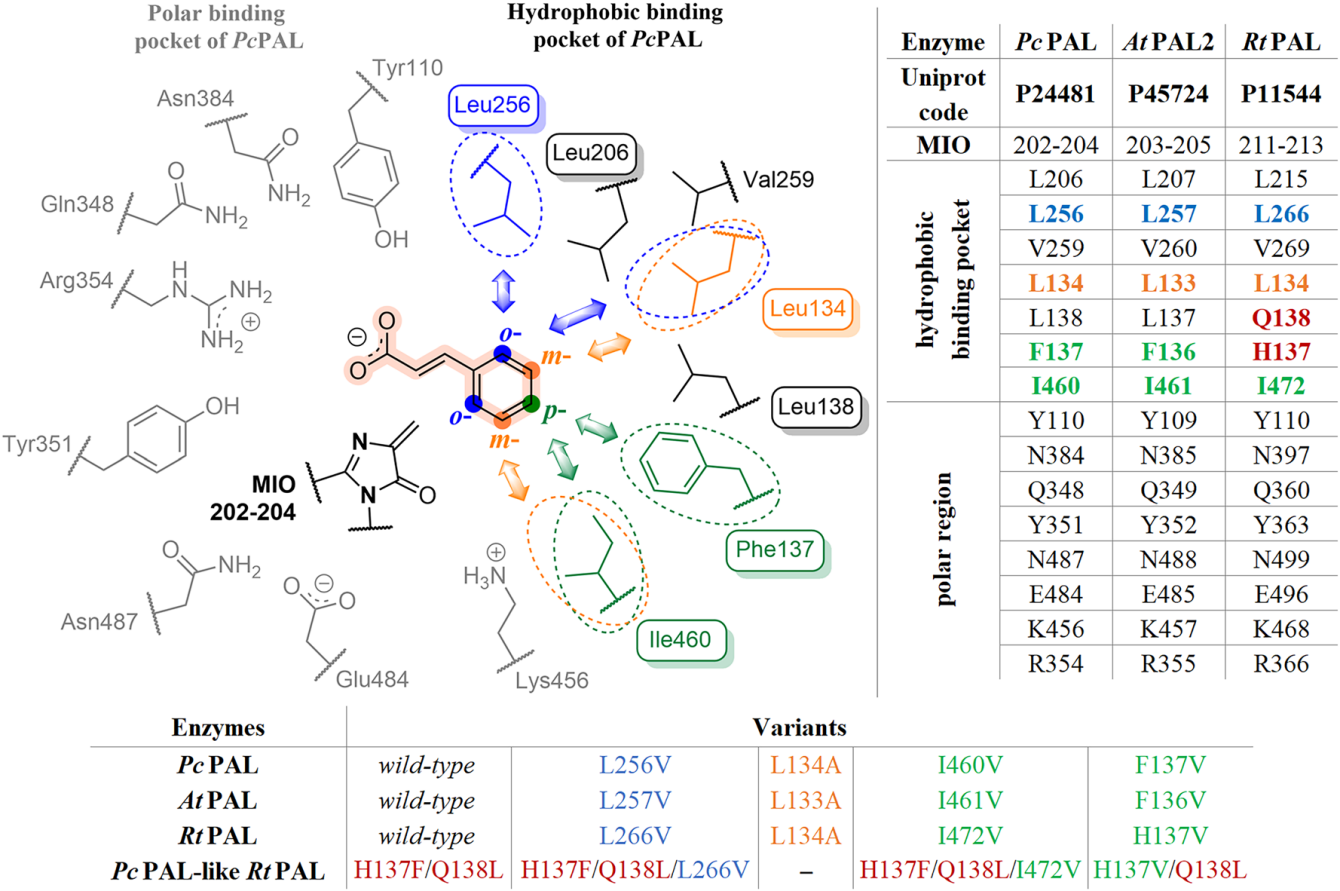
Catalytic site of *Pc*PAL, with key residues from the hydrophobic substrate-binding region marked based on their proximity^2^ to the differently positioned (*ortho*-blue, *meta*-orange and *para-*green*)* aromatic substituents of the substrates and the homologue active site residues in *Rt*PAL (P11544) and *At*PAL (P45724) based on sequence alignments with *Pc*PAL (P24481) (*right Table*) with colour-marked residues subjected to mutagenesis, generating the focused PAL variant-library (*bottom Table*).

The current state of art of the PAL mediated biotransformations revealed several synthetically useful PALs of eukaryotic (plant and yeast) or bacterial origins^1–4^. Their substrate scope has been intensively studied within the last decade, with several PALs, such as those originary from *Petroselinum crispum* (*Pc*PAL)^2,5,6^, *Anabaena variabilis* (*Av*PAL)^7,8^, *Rhodotorula glutinis* (*Rg*PAL)^9,10^, *Arabidopsis thaliana* (*At*PAL)^11^, *Planctomyces brasiliensis* (*Pb*PAL)^12^, *Kangiella koreensis* (*Kk*PAL)^13^, *Pseudozyma antarctica* (*Pza*PAL)^14^, shown to possess broad substrate scope. However, all these studies also revealed significant differences in their catalytic efficiencies towards specific substrates^2,7,11,12,14^. As example, *Pb*PAL and the recently explored AL-11 PAL^15^ transformed substrates with electron-donor substituents, previously shown to be poor substrates for other PALs, such as *Pc*PAL, *At*PAL and *Av*PAL. Comparison of the catalytic sites of PALs of different origins shows a highly conserved polar substrate binding region responsible for the fixation of substrate’s carboxyl- and NH_2_- group (Fig. 1, Fig. S1), which also embeds the catalytically essential 3,5-dihydro-5-methylene-4*H*-imidazol-4-one MIO-group^16,17^. Besides, the residues of the polar binding region form an essential H-bond network^18^ (Fig. S2). The differences within the hydrophobic substrate-binding region of PALs (Fig. 1, Fig. S1), responsible for the facile active site accommodation of the substrate’s aromatic ring, supposedly contribute to the different substrate specificities observed among aromatic ammonia lyases^2,4,19^. Interestingly, several hydrophobic active site residues, such as those corresponding to I460 and L256 of *Pc*PAL are highly conserved, while large diversification can be observed at positions homologue with 137 and 138 of *Pc*PAL (Fig. 1, Fig. S1). These later residues are well-known for their substrate-specificity modulator effect, specific polar residues (e.g. histidine) at position corresponding to 137 of *Pc*PAL provide also tyrosine ammonia-lyase (TAL) activity, such as in case of *Rg*PAL and *Rt*PAL from *Rhodotorula* sp., with reported TAL/PAL activities^20^. Histidine ammonia-lyases (HALs) show a characteristic His residue at position corresponding to 138 of *Pc*PAL^19,21^. Exploration and characterization of novel PALs from different origins is continuously expanding^12–15^ driven by the aim to find PALs of increased operational and/or thermostability, or of activity towards substrates hardly transformed by existing PALs.

Protein engineering efforts on PALs of different origins, such as *Pc*PAL^2,6,18,22^, *Av*PAL^7,23^, *Rg*PAL^24^, *Pb*PAL^12^, AL-11^15^, also focused to provide variants of expanded substrate scope or increased operational and thermal stability. Diversification at positions 137 and 138 has been performed at several PALs, such as *Pc*PAL^2,6,18^, *Av*PAL^7,8,23^, *Rt*PAL^24^ and *Pb*PAL^12^, and provided variants of improved catalytic performance, with modifications especially at analogue positions of 137 of *Pc*PAL, highlighting the substrate specificity modulator effect of this residue. Our recent mutational analysis of the hydrophobic binding pocket of *Pc*PAL^2^, revealed other specificity modulator active site residues and depicted their specific interaction with the differently positioned *ortho-*, *meta-*, *para-* substituents of the non-natural substrates, thus providing excellent tool for the rational protein engineering of *Pc*PAL. Considering the differences in the hydrophobic substrate binding pocket of PALs of different origins, that strongly influence their substrate scope, the general validity of our recently developed rational engineering strategy among PALs of diverse origins, hence of diverse substrate scope, should be assessed. This might provide a desirable general rational design strategy among PALs, allowing facile development of substrate-tailored PALs of various origins. Accordingly, we tested whether the mutational strategy developed for *Pc*PAL applies to other PALs, such as *At*PAL and *Rt*PAL, of different sequence similarities to *Pc*PAL, *At*PAL possessing high, 81% sequence identity and identical catalytic site with *Pc*PAL, while *Rt*PAL shares lower, 38% sequence identity with *Pc*PAL, and contains specific ‘TAL-activity provider’ His and Gln residues at positions analogue to 137 and 138 of *Pc*PAL (Fig. 1). Notable, that *wild-type At*PAL and *Pc*PAL show very similar catalytic efficiencies (k_cat_), while in comparison *Rt*PAL shows ~ twofold increased k_cat_ values, but lower specificity constants (k_cat_/K_M_) in the natural PAL-reaction (deamination of l-Phe) and also within the reverse ammonia addition route of *trans*-cinnamic acid^11^.

## Results and discussion

### Mutant library generation and enzyme activity screens

Recent mapping of the hydrophobic binding pocket of *Pc*PAL, revealed mutant variants, obtained by mutagenesis of residues L256, L134, F137 and I460, of enhanced catalytic activity to *ortho*-, *meta*-, *para*- substituted substrates, respectively^2^. Accordingly, the homologues of all four substrate specificity modulating residues were similarly replaced in *At*PAL and *Rt*PAL (Fig. 1), initially obtaining four *At*PAL variants (L257V, L133A, F136V and I461V) and four *Rt*PAL variants (L266V, L134A, H137V and I472V). Notable, that residue H137 of *Rt*PAL, homologue of F137 of *Pc*PAL, is most probably involved in H-bonding with Q138, homologue of L138 of *Pc*PAL. Since H137V *Rt*PAL variant showed reduced activity within the activity screens, we presumed that the presence of residue Q138 within a complete hydrophobic active site environment is non-favourable. Thus, the mutational strategy was adapted, and within the ‘*Pc*PAL-like’ *Rt*PAL variants, besides the specific mutation of residues involved in aromatic substituent accommodation, additional mutations H137F and Q138L have also been included. Thus, *Rt*PAL variant H137F/Q138L resembles the catalytic site of *wt*-*Pc*PAL, variants H137F/Q138L/L266V and H137F/Q138L/I472V being homologues to L256V and I460V *Pc*PAL, respectively, and variant H137V/Q138L *Rt*PAL to F137V *Pc*PAL variant. Despite our efforts, including different PCR protocols for mutagenesis, *Rt*PAL variant L134A/H137F/Q138L resembling the catalytic site of L134A *Pc*PAL could not be obtained, thus only L134A *Rt*PAL was employed within the activity tests. The slight variations in thermal unfolding temperatures (T_m_) of the purified enzyme variants indicated that mutations did not affect the protein folding (Figs. S3, S4 and Table S2), the only significant modification being observed in case of variant H137F/Q138L/I472V *Rt*PAL, with T_m_ value decreased with ~ 6 °C compared to the *wild-type Rt*PAL.

### Activity assessments of the *Pc*PAL, *At*PAL and *Rt*PAL variant library

**Figure 2:**
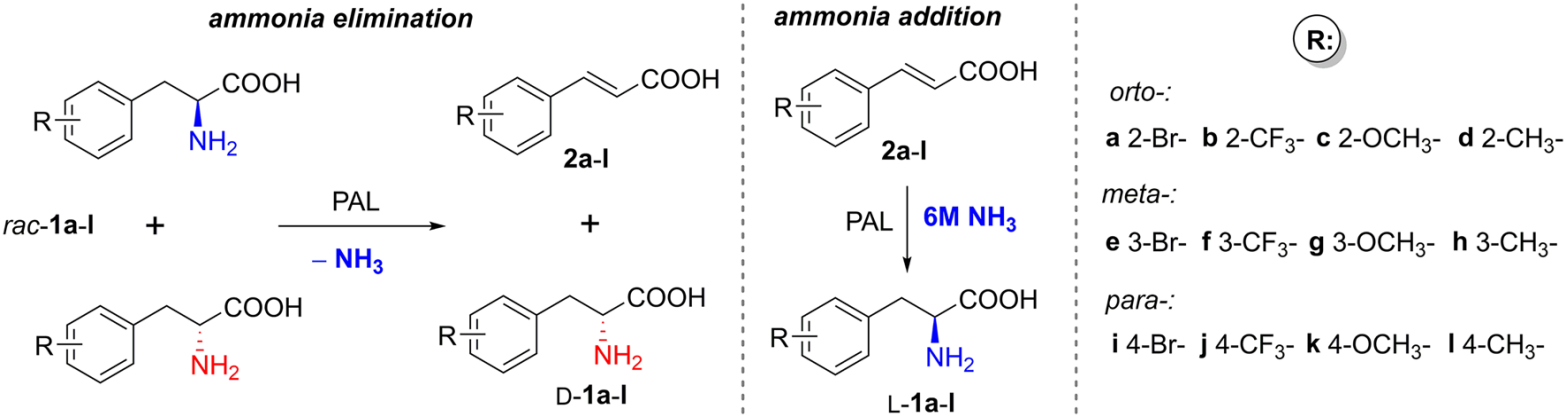
The activity assessment of the *Rt*PAL, *At*PAL and *Pc*PAL variant library within the deamination and amination reactions of phenylalanines *rac*-**1a–l** and cinnamic acids **2a–l**, monosubstituted at *ortho-, meta-, para-* positions of their aromatic ring.

The activities of the obtained *Rt*PAL and *At*PAL variants were assessed and compared with those of the corresponding *Pc*PAL variants within the deamination and amination reactions of phenylalanines and cinnamic acids, monosubstituted with both electron-donating (− CH_3_, − OCH_3_) and electron-withdrawing (− CF_3_, − Br) groups at all positions (*ortho, meta, para*) of their aromatic ring (Fig. 2). Both reaction routes have been tested by whole-cell PAL-biocatalysts mediated biotransformations of substrates *rac*-**1a–l** and **2a–l**, monitoring the conversions, while for the ammonia eliminations enzyme kinetic parameters (K_M_ and k_cat_), based on initial velocity measurements using purified enzymes, were also assessed. During enzyme kinetic measurements for the ammonia additions, factors such as (i) the high ammonia concentration of the reaction buffer leading to increased background absorbance, (ii) the high extinction coefficient of the cinnamic acid derivatives, hindering the use of large substrate concentrations, (iii) the overlapping absorbance spectra of the cinnamic acid and phenylalanine counterparts, provided high standard deviations, low reproducibility and/or incomplete Michaelis–Menten curves.

#### Activity assessments for *ortho*-substituted substrates

Generally, within the whole-cell mediated biotransformations of *ortho*-substituted substrates **1,2a–d**, mutations L257V, L133A in case of *At*PAL and mutation L266V in case of *Rt*PAL provided similar enhancement of the conversion-based enzyme activity, relatively to their *wild-type* variants, as the one reported for L256V *Pc*PAL^2^ (Table 1). Additional general tendency can be observed among the results obtained with *wild-type* PALs, *At*PAL outperforming in terms of conversion the corresponding *Pc*PAL, while *wt*-*Rt*PAL provided the lower conversions in both reaction routes (Table 1).

**Table 1:**
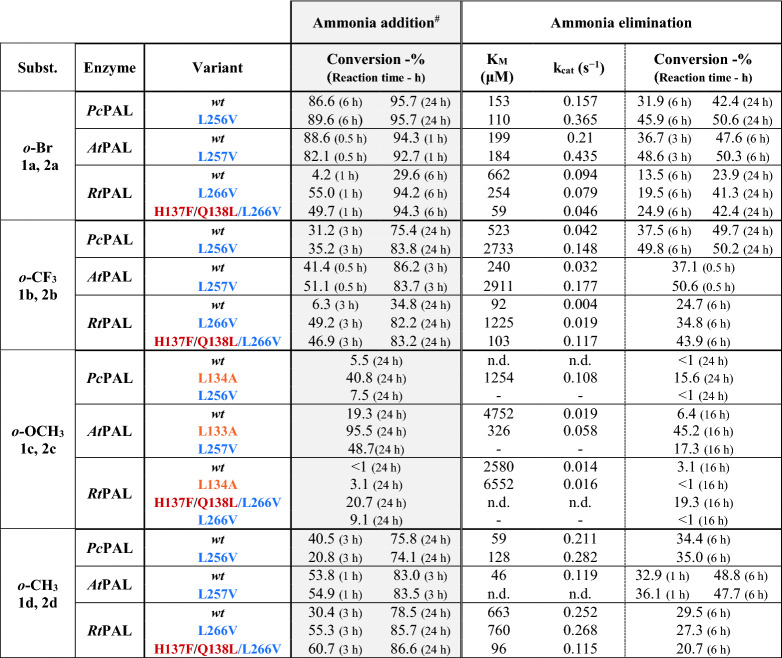
Activity assessment of the different PAL variants within the ammonia addition and ammonia elimination reactions of *ortho*-substituted cinnamic acids **2a–d** and *rac*-phenylalanines **1a–d**, respectively.

*n.d.* not determinable, during enzyme kinetics the non-linear range of the Michaelis–Menten curve was not obtained using substrate concentration allowed by the solubility of the tested compounds.

^#^During the ammonia additions the enantiomeric excess of the obtained l-phenylalanine analogues was also monitored, in all cases *ee* > 99% have been obtained.

**Figure 3:**
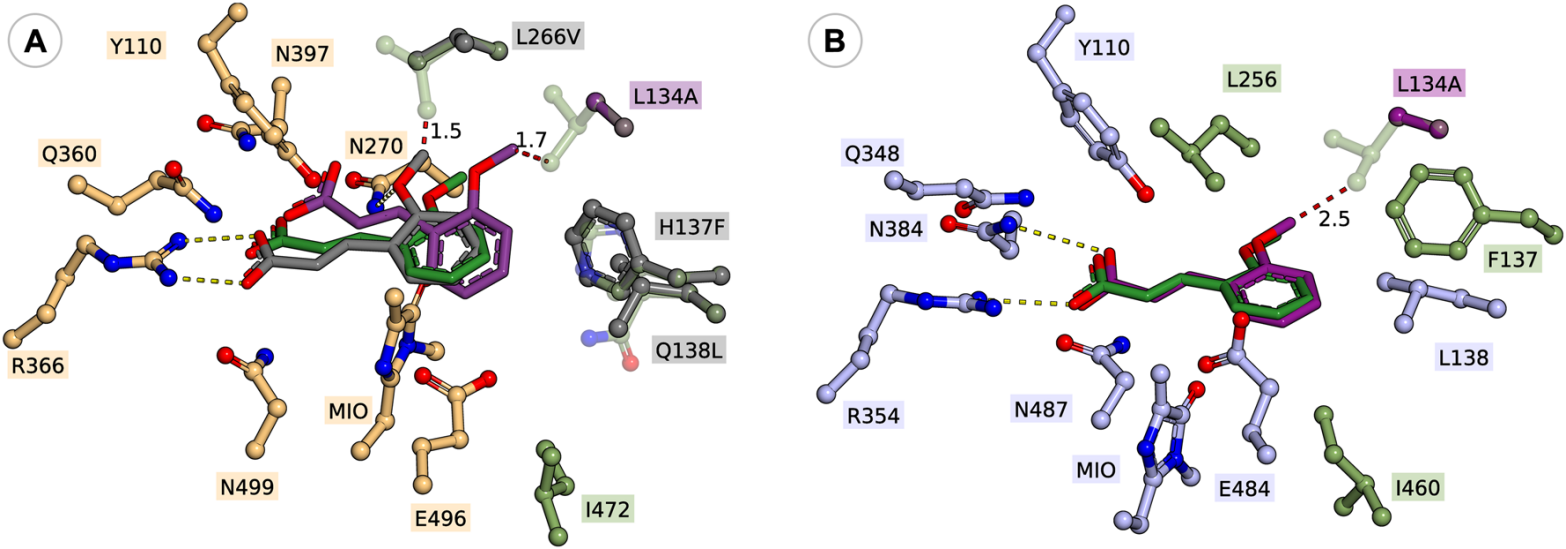
2-Methoxycinnamic acid **2c** docked into the active site of: (**A**) *wt*-*Rt*PAL (green, − 4.2 kcal/mol), L134A *Rt*PAL (purple, − 6.9 kcal/mol), and H137F/Q138L/L266V *Rt*PAL (grey, − 8 kcal/mol); (**B**) *wt*-*Pc*PAL (green, − 7.3 kcal/mol) and L134A *Pc*PAL (purple, − 7.7 kcal/mol). Steric clashes between the *ortho*-methoxy group and side chains of residues L266 and L134, respectively, are highlighted with red dashed lines. The modified residues and the active site orientation of **2c** within the corresponding PAL variant is marked with similar colour.

More detailed, in case of *o*-Br-substituted substrates excellent, equilibrium-approaching conversions are obtained with the *wild-type Pc*PAL (87% for **2a** and 42% for **1a** after 6 h and 24 h reaction times) and *At*PAL (94% for **2a** after 1 h reaction time and 48% for **1a** after 6 h reaction time), thus the increased catalytic efficiency of variants L256V *Pc*PAL and L257V *At*PAL is less reflected within the conversion-based enzyme activities. However, the 2.3- and 2.1-fold increased k_cat_ values, comparatively to the *wt*-variant’s (Table 1), support the beneficial effect of the mutations. Similar behaviour can be observed in case of substrates **1b** and **2b**, with high conversions of similar range being registered for both *wt-* or mutant variants of *Pc*/*At*PAL, but 3.5- and 5.5-fold increased catalytic efficiencies (k_cat_) of the corresponding L256V and L257V variants. *At*PALs provided stationary conversions of ~ 86% for **2b** and ~ 50% for *rac*-**1b** within significantly shorter reaction times of 3 h and 30 min, respectively, in comparison with similar conversions obtained only after 24 h reaction times using *Pc*PALs. Interestingly, while in case of **1a** the K_M_ value was not significantly altered upon mutations analogue to L256V, in case of **1b** the mutation resulted in highly decreased substrate affinity (increased K_M_ values) for all three PALs of different origin, supporting a more relaxed accommodation of **1b** within the modified active site. *Wild-type Rt*PAL in case of both substrates **2a**, **2b** provided only moderate conversion of 30% (6 h) and 35% (24 h), respectively, thus the beneficial effect of mutation L266V was clearly visible also within the conversion-based enzyme activity, with 94% (6 h) and 82% (24 h) conversion for **2a** and **2b**, respectively. In case of *o*-OCH_3_- substituted substrates **1c, 2c**, the beneficial effect of mutations analogue with L134A from *Pc*PAL was also observed in case of *At*PAL, where the corresponding L133A variant provided high conversions of 95% and 3.1-fold increased k_cat_ values. In case of L134A, but also *wild-type Rt*PAL, very low/no conversions of < 1–3% were detected, while enzyme kinetics also revealed low initial velocities and substrate affinities. Interestingly, in this case the ‘*Pc*PAL-like’ *Rt*PAL L266V variant, with mutations H137F/Q138L/L266V, provided increased conversions of 19% for **1c** and 17% for **2c** after 16 h reaction time. Indeed, in this particular case, due to the bend caused by the oxygen atom of the *o*-OCH_3_ substituent, the methyl group positions between residues 134 and 266 (Fig. 3A,B), while the increased hydrophobicity induced by mutation H137F and Q138L most probably facilitates the accommodation of the substrate’s aromatic moiety. In accordance with the experimental results, both flexible and rigid docking of **2c** within the active sites of *wild-type*, L134A and H137F/Q138L/L266V *Rt*PAL revealed substrate orientations of significantly lower energy for both mutant variants in comparison with those obtained for the *wild-type Rt*PAL (Fig. 3A). In case of *o*-CH_3_-substituted substrates **1d, 2d** the *wild-type* variants of all three PALs provided high conversion in both reaction routes, the best performer *At*PAL reaching in shortest reaction time of 3 h 83% conversion of **2a**. Similarly to the case of the *o*-Br-substituted substrates **1a**, **2a**, the increased catalytic efficiency of the variants bearing mutations analogue to L256V of *Pc*PAL is supported by their increased k_cat_ values in comparison to their *wild-type* variants. The less significant, only 1.1–1.3-fold increase in k_*cat*_ values, than in case of **1a–c**, is expectable based on the smallest sterical requirement of the methyl group, which seemingly, when *ortho*-positioned on the substrate, is favourably accommodated within the active site of all PAL variants.

#### Activity assessments for *meta*-substituted substrates

**Figure 4:**
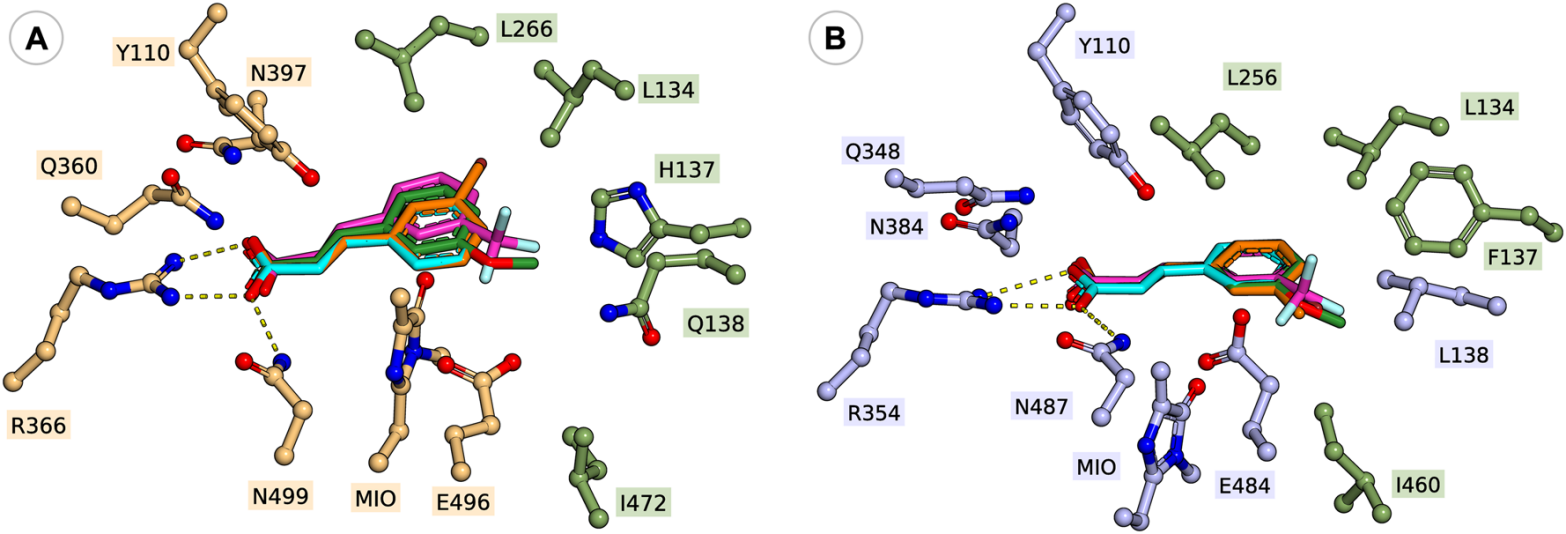
Active orientations of *meta*-substituted substrates *m*-Br-cinnamic acid **2e** (cyan), *m*-CF_3_-cinnamic acid **2f** (magenta), *m*-OCH_3_-cinnamic acid **2g** (green) and *m*-CH_3_-cinnamic acid **2h** (orange) within (**A**) *wt*-*Rt*PAL and (**B**) *wt*-*Pc*PAL.

Interestingly, in case of phenylalanine/cinnamic acid analogues with substituents in *meta*-position **1e, 1f, 1h** and **2e, 2f, 2h**, the *wild-type* variant *Rt*PAL showed superior catalytic efficiency in comparison to *wild-type Pc*PAL and *At*PAL, supported by its higher k_cat_ values within the ammonia eliminations of **1e**, **1f**, **1h** or conversion values in both reaction routes of *m*-CF_3_- and *m*-Me-substituted substrates (Table 2). The case of *m*-methoxy-substituted substrates **1g**, **2g** acts again as an exception, where *Rt*PAL shows significantly lower, ~ 18% conversion within the ammonia addition, while 52% and 32% conversion are registered with *At*PAL and *Pc*PAL, respectively. Within the two presumed active orientations of the *meta*-substituents (Fig. 1), in case of *Rt*PAL besides the conserved L134 and I472 residues, polar residues Q138, H137 also appear, suggesting their favourable interaction with the polar CF_3_- and Br- substituents of **1e**,** 2e** and **1f**,** 2f**, that most probably contributes towards the superior activity of *Rt*PAL. Computational results revealed that the substrate orientations exposing the *meta*-substituent towards residue I460 are energetically favoured in case of *wild-type Pc*PAL, while for *wild-type Rt*PAL the presence of polar residues Q138, H137 shifts the active site orientation of the *meta*-substituent from those observed for *Pc*PAL, the orientations towards residues L134 showing close or even lower energies than those pointing towards I472 of *Rt*PAL (Fig. 4, Fig. S5, Table S7).

**Table 2:**
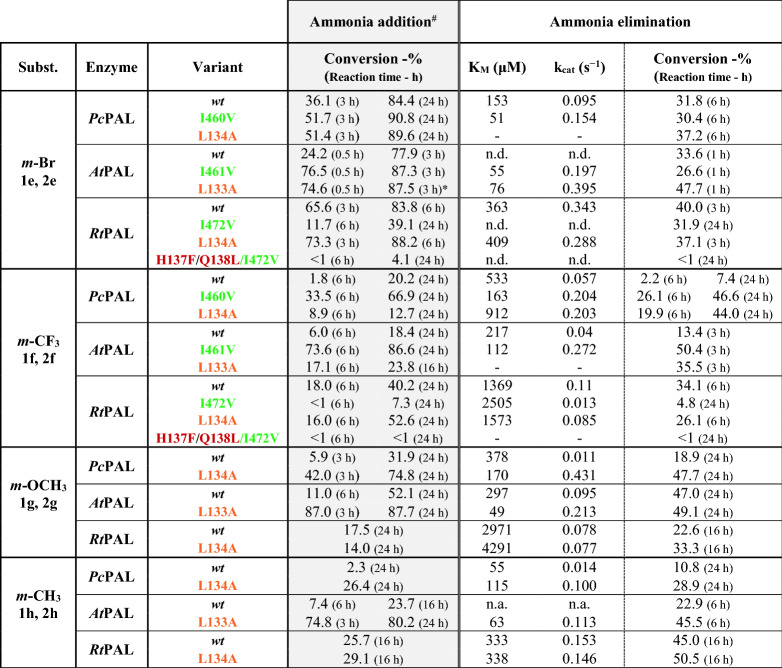
Activity assessment of the different PAL variants within the ammonia addition and ammonia elimination reactions of *meta*-substituted cinnamic acids **2e**–**h** and *rac*-phenylalanines **1e**–**h**, respectively.

*n.d.* not determinable, during enzyme kinetics the non-linear range of the Michaelis–Menten curve was not obtained using substrate concentration allowed by the solubility of the tested compounds.

*n.a.* no activity detected.

“–” no determination/measurement was performed.

^**#**^During the ammonia additions the enantiomeric excess of the obtained l-phenylalanine analogues was also monitored, in all cases *ee* > 99% have been obtained.

*Except in case of the l-**1e** (*ee* = 93.6%) produced within the ammonia addition reaction of **2e** catalyzed by L133A *At*PAL.

The beneficial effect of the mutational strategy explored at *Pc*PAL for the increased enzyme activity towards *m*-substituted substrates, was perfectly retained in case of *At*PAL, where mutations L133A and I461V provided significantly increased k_cat_ and conversion values in case of all substrates. While the results were expected in the frame of identical architecture of the two catalytic sites of *At*PAL and *Pc*PAL, in most of the cases *At*PAL variants I461V and L133A showed superior catalytic properties (higher k_cat_ values and higher conversions in shorter reaction time), than their corresponding *Pc*PAL homologues (Table 2). In case of *Rt*PAL, deciphering the beneficial effect of homologue mutations L134A and I472V was hindered by the low activity of I472V *Rt*PAL and its “*Pc*PAL-like” homologue, H137F/Q138L/I472V variant, while the above discussed high activity of the *wild-type Rt*PAL supports that it also represents an optimized variant for *meta*-substituted substrates, substrate orientations exposing the aromatic substituents towards residue L134 being favoured. Accordingly, variant L134A provided conversion and kinetic data close to those observed for *wt-Rt*PAL for all tested substrates, while the unsuccessful mutagenesis in case of its *Pc*PAL-like L134A/H137F/Q138L variant didn’t allow testing the combined effect of replacing polar residues H137, Q138 and *meta*-substituted substrate-modulator residue L134. Moreover, variants including mutation I472V used as purified proteins were completely inactive within kinetic measurements, also providing very low conversion when used as whole-cell biocatalysts in biotransformations of **1e, 1f** and **2e, 2f**. Their thermal denaturing profile (Fig. S4) reveals their lowered thermal stability, similar to those reported for I460A *Pc*PAL variant, for which we supposed that the mutation-induced, non-favourable water-accessibility of the catalytic site is responsible for the activity loss^6^. Notable, that variant I472V *Rt*PAL and its ‘*Pc*PAL-like’ homologue H137F/Q138L/I472V were also inactive within the biotransformations of *p*-substituted substrates (Table 3).

#### Activity assessments for *para*-substituted substrates

In case of *para*-substituted phenylalanines **1j**, **1k**,**1l** and cinnamic acids **2i**–**2l** very low (< 10%) or no conversion was detected when using *wild-type Pc*PAL and *Rt*PAL variants, in accordance with the reported steric clashes between the *p*-substituent and active site residues^2,6^. Interestingly, *wt*-*At*PAL afforded close to maximum conversion of all *para*-substituted phenylalanines, except for *p*-OCH_3_-phenylalanine, where similarly to *Pc/Rt*PALs, low conversion of 14% and k_cat_ value of 0.007 s^−1^ were obtained within the ammonia elimination of *rac*-**1k** and no conversion within the ammonia addition to **2k**.

Related to the effect of the mutational strategy, we observed that in case of *At*PAL variants I461V and F136V, similarly as in case of *Pc*PAL, provided important conversion and activity enhancements for all substrates **1i**–**2l** and **2i**–**l**. Accordingly, while in case of *p*-Br- and *p*-CF_3_-substituted substrates the mutation-induced increase in the conversions is less significant, due to the well-performing *wild-type* variant, the 2.9-fold and 3.4-fold increased k_cat_ values of variant I461V for **1i** and **1j** support the beneficial effect of the mutation. In case of substrates **1k**, **1l** and **2k**, **2l**, *p*-substituted with the electron-donating -OCH_3_ and -CH_3_ groups, the superior catalytic efficiency of I461V variant to *wt*-*At*PAL is also resembled within the highly increased conversion values. While mutation F136V of *At*PAL also induced significant increase in the conversions of all substrates, in case of substrates **1i**, **1j** and **2i**, **2j** even surpassing the conversions registered with I461V variant, however the enantiomeric excess (*ee*) of the l-phenylalanines **1i**, **1j** and **1 k** produced within the ammonia additions, were of lower value (*ee* of 92%, 83% and 97%, respectively) in comparison with the highly enantiopure forms (*ee* > 99%) produced by *wt*- and I461V variant. This is in accordance with the results from *Pc*PAL, where mutation F137V also decreased the enantioselectivity of the enzyme with *ee* values of 97% and 82% being obtained for l-1i and l-1j, respectively.

**Table 3:**
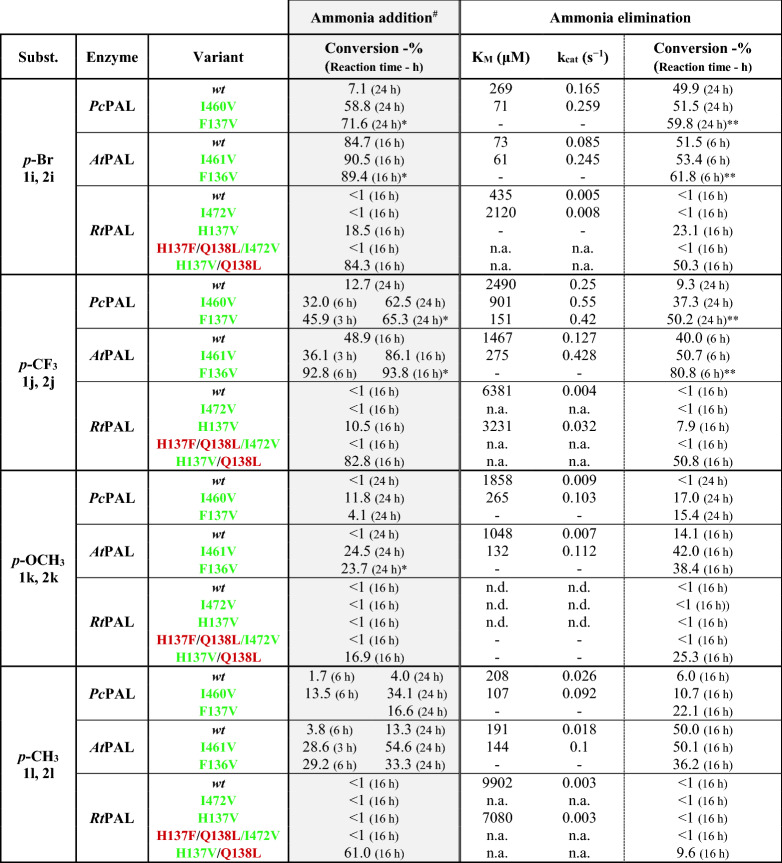
Activity assessment of the different PAL variants within the ammonia addition and ammonia elimination reactions of *para*-substituted cinnamic acids **2i**–**l** and *rac*-phenylalanines **1i**–**l**, respectively.

*n.d.* not determinable, during enzyme kinetics the non-linear range of the Michaelis–Menten curve was not obtained using substrate concentration allowed by the solubility of the tested compounds; *n.a.* no activity detected; “-” no determination/measurement was performed.

^**#**^During the ammonia additions the enantiomeric excess of the obtained l-phenylalanine analogues was also monitored, in all cases *ee* > 99% have been obtained, with marked exceptions.

*1. In case of **2i: Pc**PAL F137V variant provided l-1i with *ee* = 97%; *At*PAL F136V variant provided l-1i with *ee* = 93%; **2**. in case of **2j**: *Pc*PAL F137V variant provided l-1j with *ee* = 82%; *At*PAL F136V variant provided l-1j with *ee* = 83%; **3**. in case of **2k**: *At*PAL F136V variant provided l-1k with *ee* = 97%.

**During the kinetic resolution-type ammonia eliminations in case of high enantioselectivity the maximal conversion values of *rac*-phenylalanines is 50%, conversions exceeding this value, support the low enantioselectivity of the process.

**Figure 5:**
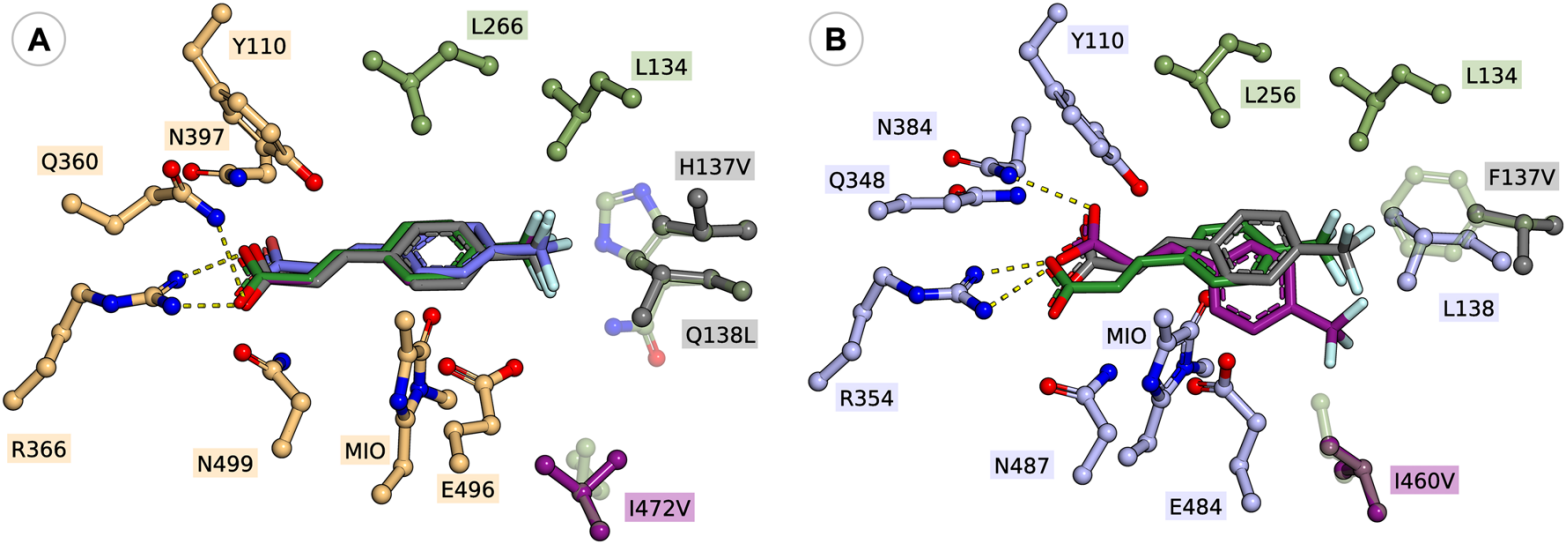
Active orientations of 4-(trifluoromethyl) cinnamic acid **2j** within (**A**) *wt*-*Rt*PAL (green, − 5.5 kcal/mol), H137V *Rt*PAL (indigo, − 6.9 kcal/mol), H137V/Q138L *Rt*PAL (grey, − 7.5 kcal/mol), and I472V *Rt*PAL (purple, − 5.5 kcal/mol) and (**B**) *wt*-*Pc*PAL (green, − 4.8 kcal/mol), F137V *Pc*PAL (grey, − 8.9 kcal/mol), and I460V *Pc*PAL (purple, − 6.4 kcal/mol). The modified residues and the active site orientation of **2j** within the corresponding variant is marked with similar colour.

*Rt*PAL, in general, proved to be inefficient for the transformation of *para*-substituted amino acids, while the destabilization effect of mutation residue I472V, as described in case of *meta*-substituted substrates, resulted in no detectable activity. Instead, the mutation H137V, provided minor to moderate conversion increase of 7.9–23.1%, in case of *p*-Br- and *p*-CF_3_-substituted substrates **1i**, **1j** and **2i**, **2j**, where the beneficial effect of the mutation is also supported by the significantly increased k_cat_ values. The lower catalytic efficiency of H137V *Rt*PAL, reflected in significantly lower conversion values, in comparison to its homologue variants F136V *At*PAL and F137V *Pc*PAL, might result from the presence of polar Q138 residue in the proximity of the hydrophobic, mutated V137 residue (Fig. 5), supported by the increased conversions provided by the ‘*Pc*PAL-like’ H137V/Q138L *Rt*PAL, approximating the conversions registered with the homologue *At*/*Pc*-PAL variants.

**Figure 6:**
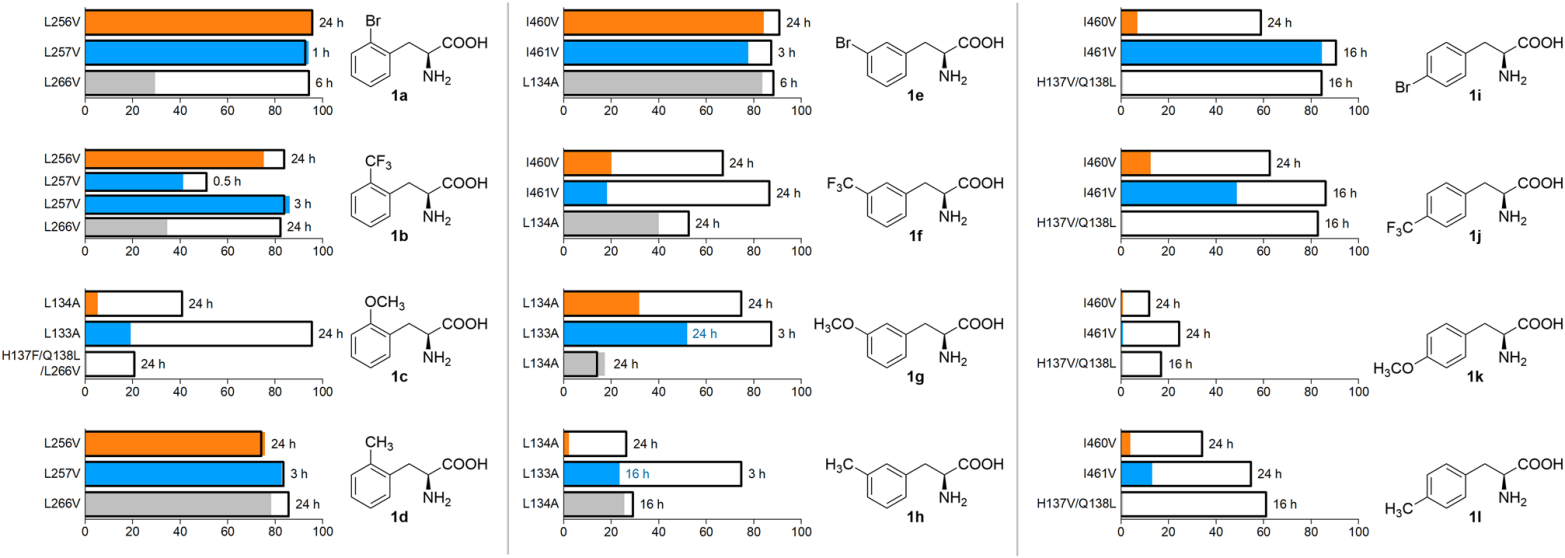
Conversion into l-phenylalanines l-**1a–l** obtained within the ammonia additions reactions catalyzed by the *wild-type Pc*PAL (orange), *At*PAL (blue) and *Rt*PAL (grey) overlayed with the conversions provided by their best performing mutants (marked with non-filled boxes overlayed with the coloured lanes, representing the conversions of *wild-type* variants), evidencing the conversion-based activity increase provided by the mutations (white zone of each lane-box).

Considering the above described conservation of the catalytic efficiency-enhancing effect (Fig. 6) of the mutational strategy developed for *Pc*PAL, in case of *At*PAL (81% sequence identity and identical catalytic site with *Pc*PAL) and *Rt*PAL (38% sequence identity and TAL-activity providing catalytic site, containing H137, Q138 residues at positions analogue to F137, L138 of *Pc*PAL) the general applicability of the rational design strategy among PALs is supported. Notable, that in case of *Rt*PAL, besides the modification of the substrate specificity-modulator residues, replacement of residue Q138, in proximity of position 137, to hydrophobic residues, further enhanced the catalytic properties of H137V *Rt*PAL, supporting that the mutational strategy is adaptable for further additional mutations based on simple rational considerations, allowing facile development of substrate-tailored PALs of various origins. Despite the identical catalytic site residues of *At*PAL and *Pc*PAL, in several cases *At*PAL variants in comparison with the corresponding *Pc*PAL variants, showed higher catalytic efficiencies/conversions (Fig. 6), highlighting that besides active site residues, other structural elements also determine the different enzyme activities/substrate specificities of PALs of different origins. Besides, the mutational approach revealed several PAL variants, such as L133A *At*PAL, I461V *At*PAL, L266V and H137V/Q138L *Rt*PAL, which in comparison with their previously reported^2^ *Pc*PAL homologues, possess enhanced catalytic efficiency within the various ammonia additions producing valuable l-phenylalanines (Fig. 6).

## Experimental part

### Site-directed mutagenesis

The codon optimized genes encoding PALs from *Arabidopsis thaliana* and *Rhodosporidium toruloides* were obtained through the synthesis services of GenScript, followed by their cloning into pET19b vector (using XhoI and Bpu1102I cloning sites for *Rt*PAL and XhoI and NdeI cloning sites for *At*PAL). The site-directed mutagenesis was performed following the protocol described by Naismith and Liu^25^, using as template the pET-19b vector harbouring the gene encoding PALs from *Arabidopsis thaliana* and *Rhodosporidium toruloides,* respectively. Using as homology model the active site of *Pc*PAL^6^ (PDB ID 1W27^26^), several residues from the hydrophobic binding pocket were selected for point mutations, namely L133/L134 (to A), F136/H137 (to V), L257/L266 (to V) and I461/I472 (to V). New MIO-enzyme libraries were created at these positions and screened for activity towards the substrates. The primers used within the mutagenesis are listed in Table S1.

### Protein expression, purification

Expression, isolation and purification of *wild-type Rt*PAL and its mutant variants (L134A, H137V, L266V, I472V, H137F/Q138L/L266V, H137F/Q138L/I472V, H137F/Q138L, H137V/Q138L) was performed according to our optimized protocol via immobilized affinity chromatography (IMAC)^27^. In case of *At*PAL (*wt-* and L133A, F136V, L257V, I461V mutants), precultures were prepared at 37 °C, 200 rpm, overnight in 50 mL LB (Luria Bertani) medium supplemented with carbenicillin (50 μg/mL) and chloramphenicol (30 μg/mL) from glycerol stocks of *E.coli* Rosetta (DE3)plysS cells harbouring the pET-19b vector carrying the *wt-* or mutant *atpal* gene. 2% (v/v) from the starter culture was used to inoculate 2 × 500 mL LB medium in 2 L flasks. The OD_600_ was monitored and when a value of 0.45 was reached, the temperature was lowered from 37 to 25 °C, and the shaking continued till an OD_600_ value of 0.6–0.8, when PAL expression was induced via IPTG (0.5 mM final concentration). The cell growth continued at 25 °C, 200 rpm for another 6 h, when cells were harvested by centrifugation at 4000 rpm (1751×*g*), 4 °C for 20 min. The supernatant was discarded and the cell pellet was stored at − 20 °C until further use or processed immediately using the optimized protein isolation protocol as described for *Pc*PAL^27^.

### Thermal unfolding profile of purified proteins

The thermal unfolding of all PALs was determined by nanoscale differential scanning fluorimetry measurements, using Prometheus NT.48 nanoDSF instrument (NanoTemper Technologies, München, Germany). PAL variants were diluted with 20 mM Tris, 120 mM NaCl pH 8.8 buffer to a final concentration of 1 mg/mL. 10 μL of each sample were loaded into UV capillaries (NanoTemper Technologies) and unfolding of PAL enzymes was detected during heating in a linear thermal ramp of 1.5 °C/min between 20 and 95 °C, with an excitation power of 70%. Data analysis was performed using NT Melting Control software and melting temperature (T_m_) was determined by fitting the experimental data using a polynomial function, in which the maximum slope is indicated by the peak of its first derivative (F350/F330). All measurements were performed in triplicate (Figs. S3, S4 and Table S2).

### Preparation of whole-cell PAL biocatalysts

The overnight precultures were prepared in 20 mL LB (Luria Bertani) medium supplemented with carbenicillin (50 μg/mL) and chloramphenicol (30 μg/mL) in 100 mL Erlenmeyer flasks, being inoculated with glycerol stocks of *E. coli* Rosetta (DE3) pLysS cells harboring the pET-19b vector carrying the *wt* or mutant *atpal* or *rtpal* gene, followed by incubation at 37 °C and shaking at 200 rpm. 2% (v/v) of the overnight culture was used to inoculate 50 mL LB medium. Cultures were grown at 37 °C, 200 rpm until OD_600_ reached 0.6–0.8 (approx. 3 h), when enzyme production was induced via the addition of 0.5 mM IPTG (final concentration), and the cell growth was maintained at 20 °C, 200 rpm, overnight (approx. 17 h). The final OD_600_ was measured for each mutant variant and *wild-type* PAL. The culture volumes required for the biotransformation screenings were harvested by centrifugation in 1.5 mL polypropylene tubes for 10 min at 13,300 rpm (12,000×*g*). The required volume of bacterial culture, providing the amount of whole-cell pellet needed was calculated considering the volume of the reactions, the whole-cell biocatalysts concentration (with fixed cell density OD_600_ of ~ 2)^2,22^ and the final OD_600_ value of the induced cells. The harvested cells were washed with 500 μL PBS buffer (20 mM phosphate, 150 mM NaCl, pH 8.0) (13,300 rpm, 12,000×*g*, 10 min) and stored at − 20 °C until further use.

### Analytical scale ammonia addition and elimination reactions

The bacterial pellet of PAL-biocatalysts (prepared as described above, in 1.5 mL polypropylene tubes) was resuspended to an OD_600_ of ~ 2, in 500 µL substrate solution (2 mM cinnamic acids **2a–l** or 2 mM racemic amino acids *rac-1a–l*) prepared in 6 M NH_4_OH buffer pH 10 adjusted with CO_2_ (in case of ammonia addition) or 20 mM Tris.HCl, 120 mM NaCl buffer, pH 8.8 (in case of ammonia elimination). The reaction mixtures were incubated at 30 °C, 250 rpm. Reaction samples were taken after 3, 6, 16, and 24 h and quenched by adding an equal volume of MeOH, vortexed and centrifuged (13,400 rpm, 12,000 *g*×, 10 min). The supernatant was filtered through a 0.22 μm nylon membrane filter prior to analysis by HPLC. In order to determine the conversions values, a Gemini NX-C18 column (150 × 4.5 mm; 5 µm) was chosen, using as mobile phase: A: NH_4_OH buffer (0.1 M, pH 9.0)/B: MeOH, with a flow rate of 1.0 mL/min. The enantiomeric excess values were determined by chiral HPLC separations, using Crownpak CR-I (+) chiral column (150 × 3 mm; 5 µm) and HClO_4_ (pH = 1.5)/acetonitrile as mobile phase at a flow rate of 0.4 mL/min. HPLC methods and response factors used for the conversion value determinations, as well as retention times of the enantiomers of ***rac-1a–l*** can be consulted in our previous reports^2,6^. All analytical scale biotransformations were performed in duplicates, while during the initial activity screens using a significantly sized reaction-subset the HPLC analysis have been performed for all samples within the duplicate set (see details in Supporting information, Chapter 6, Table S3).

### Enzyme kinetics

The initial enzyme activities were spectrophotometrically determined, using a Tecan Infinite Spark 10 M microplate reader and Corning 96-well Clear Flat Bottom UV-Transparent microplates. The kinetic measurements were performed in triplicate at 30 °C by monitoring the production of *trans*-cinnamic acid analogues **2a–l** at 290 nm (wavelength where the corresponding amino acids ***rac-1a–l*** showed no absorption), using substrate concentrations of 0.1–20 mM of **1a–l**, 100 mM Tris.HCl, 120 mM NaCl (pH 8.8) as buffer and purified PAL variants at fixed enzyme concentration of 0.322 μM. Kinetic constants (K_M_, v_max_) were obtained from the Michaelis–Menten curves by non-linear fitting. Standard deviations for the determined kinetic parameters are given within Tables S4–S6 (Supporting information).

### Computational studies

The ground state geometries of the monosubstituted cinnamic acid derivatives **2a–l** were obtained by calculations based on the density functional theory, performed using the Gaussian 09 software^28^ by employing the B3LYP density functional and the 6-31G(d,p) basis set. Geometry optimizations were carried out in a water solvated environment using the Polarizable Continuum Model (PCM)^29^.

The molecular docking calculations were performed with the Autodock Vina software^30^, using flexible-ligand and rigid-receptor docking. The search space was defined by embedding the binding site residues and the MIO prosthetic group. In both cases the receptor grid was defined as a cubic box with the dimension of 20 Å × 20 Å × 20 Å. The exhaustiveness search parameter of Vina was increased to 100.

The crystal structure of *Pc*PAL was retrieved from Protein Data Bank entry 6F6T^31^, whereas in case of *Rt*PAL, the AlphaFold^32^ predicted model was retrieved from the UniProt database (entry P11544)^33^. The assembled tetrameric structure was submitted for minimization using the YASARA web server^34^. Although crystal structures of *Rt*PAL are available, PDB entries 1T6J and 1Y2M, both structures present the open conformation of the protein, missing the loop containing the Y110 residue, responsible for the catalytic site closure upon substrate binding.

## Conclusions

Within this study we tested the applicability of the mutational strategy developed for PAL from *Petroselinum crispum* to other PALs with the aim to provide a general rational design strategy, highly desirable for developing substrate-tailored PALs of diverse substrate scope and origins. Accordingly, *At*PAL and *Rt*PAL, both well-characterized PAL representatives, that share different sequence identity (high degree of 81%, respectively low degree of 38%) to *Pc*PAL, with *Rt*PAL known to possess dual PAL/TAL-activity, were selected for this purpose. As expected, *wild-type Rt*PAL with low sequence identity to *Pc/At*PAL, showed different substrate specificity towards the substrate library, revealing its higher catalytic efficiency towards *meta*-substituted substrates in both ammonia elimination and ammonia addition reaction routes, while the substrate specificities of *wt*-*Pc*/*At*PAL have been found very similar. However, the enzyme activity tests of the generated focused *At*PAL, *Rt*PAL, *Pc*PAL mutant library towards the mono-substituted substrates revealed that *At*PAL variants, with some exceptions, surpassed in terms of conversion and catalytic efficiency the corresponding, previously reported *Pc*PAL homologues (L134A, L256V, F137V and I460V). Since their active sites possess identical residues, the results highlight that besides active site residues, other structural elements also determine the different enzyme activities/substrate specificities of PALs. Furthermore, the activity of PAL variants tailored towards substrates of different (*ortho*-, *meta*-, *para*-) substitution pattern, revealed that the mutational approach is applicable among different PALs, resulting the expected catalytic efficiency increase towards the targeted non-natural substrates, with minor sequence alignment-based rational refinements further improving its efficacy. Accordingly, in case of *Rt*PAL, besides the modification of the substrate specificity modulator residues L266V, L134A, F137V and I472V, replacement of residue Q138, in proximity of mutated position 137, to hydrophobic residues, further enhanced the catalytic properties of *Rt*PAL variants. In this context, the study paves the way and contributes for the development of the general rational design strategy among the PAL (E.C. 4.3.1.24) and PAL/TAL families (E.C. 4.3.1.25).

## Supplementary Information

Supplementary Information.

## Data Availability

The Uniprot identifiers of all protein sequences used within the alignments and experimental work and the Protein Data Bank (PDB) IDs for the protein structures used within the computational part are described within the manuscript, while other datasets used and/or analysed during the current study are available from the corresponding author on reasonable request.
