# Supplementary material for: Towards a general approach for tailoring the hydrophobic binding site of phenylalanine ammonia-lyases
Source: Sci Rep. 2022 Jun 23;12:10606. doi: 10.1038/s41598-022-14585-0 (PMC9226071; doi:10.1038/s41598-022-14585-0)
Supplement: Supplementary file 1 — Supplementary Information. [file 41598_2022_14585_MOESM1_ESM.pdf]

Electronic Supplementary Information

Towards a general approach for tailoring the hydrophobic binding site of phenylalanine ammonia-lyases

Souad Diana Tork*, Dr. Mădălina Elena Moisă*, Lilla Cserepes, Dr. Alina Filip, Dr. Levente Csaba Nagy, Dr. Florin Dan Irimie, Dr. László Csaba Bencze

Affiliation: Enzymology and Applied Biocatalysis Research Center, Faculty of Chemistry and Chemical Engineering, Babeș-Bolyai University, Arany János Street 11, RO-400028 Cluj-Napoca, Romania

Address for correspondence: Dr. László Csaba Bencze; email: laszlo.bencze@ubbcluj.ro

* These authors contributed equally to this work

Table of Contents

1. Materials 3

2. Instrumentation..3

3. Sequence alignment 3

4. Site-directed mutagenesis 6

5. Protein expression, isolation and folding characterization 7

5.1. Expression and purification of PAL variants...7

5.2. Thermal unfolding profile of PALs...7

6. HPLC monitoring of the enzymatic reactions...8

7. Enzyme kinetic measurements – standard deviations 10

8. Computational studies 13

9. References..13

1. Materials

The commercial chemicals and solvents were products of Sigma Aldrich or Alfa-Aesar. The primers used for mutagenesis were purchased from Antisel Ltd. (Romania), through DNA synthesis services of Invitrogen. IPTG, Phusion Hot Start DNA Polymerase, dNTPs, DpnI, agarose were all products of Thermo Fischer Scientific (Waltham, MA, USA). Plasmid extraction kit and ethidium bromide were purchased from Sigma-Aldrich (St. Louis, MO, USA). LB medium was purchased from Liofilchem (Roseto, Italy), protease inhibitor cocktail from Hoffman La-Roche (Basel, Switzerland), while the Ni-NTA Superflow resin used for affinity chromatography was from Qiagen (Hilden, Germany). The chemical synthesis of compounds *rac*-**1a-l** and **2a-l** was performed according to procedures from our earlier works[1].

2. Instrumentation

Kinetic measurements were performed on TECAN Spark 10M microplate reader equipped with a TE-cool module. Mastercyler proS from Eppendorf (Hamburg, Germany) was used to perform the PCR reactions. Size exclusion chromatography was performed using Superdex 5/150 and Aekta4 FPLC instrument (GE Healthcare), while differential scanning fluorimetry, nanoDSF, measurements were performed using Prometheus NT.48 instrument (NanoTemper Technologies, Munich, Germany). The ^{1}H and ^{13}C NMR spectra were recorded on Bruker (Billerica, MA, USA), while high performance liquid chromatography (HPLC) analyses were conducted with an Agilent (Santa Clara, CA, USA) 1200, 1260 and 1100 systems.

3. Sequence alignment

All sequence alignments were performed using the Clustal Omega.[2]

```
CLUSTAL O(1.2.4) multiple sequence alignment

PcPAL       GTDSYGVTTGFGATSHRR--TKQGGALQKELIRFLNAGIFGNGS---------------- 147
AtPAL       GTDSYGVTTGFGATSHRR--TKNGTALQTELIRFLNAGIFGNTK---------------- 146
PzaPAL      GESIYGINTGFGGSADSR--TANTRALQLALLQMQQCGVLPVPST-FPTGEPSSAPFALP 143
RtPAL       SMSVYGVTTGFGGSADTR--TEDAISLQKALLEHQLCGVLPSSFDSFRLGR--------- 154
RgPAL       DNSVYGVTTGFGGSADTR--TEDAISLQKALLEHQLCGVLPTSMDGFALGR--------- 160
KkPAL       DGVIYGVTTGYGDSVTTPVPVQDTHELPLHLTRFHGCG---------------------- 93
PbPAL       NERIYGITTGFGGMSDIPIPPQHVAQTQDNLLAFLSTS---------------------- 97
AvPAL       GEPIYGVTSGFGGMANVAISREQASELQTNLVWFLKTG---------------------- 111
AL-11       GEEIYGVTTLFGGMADVHVTREQLIDVQKIALWQHKST---------------------- 88
            .   **:.: :*          .
....

PcPAL       TASGDLVPLSYIAGLLTGRPNSKAVGP-----TGVILSPEEAFKLAGVEGGFFELQPKEG 255
AtPAL       TASGDLVPLSYIAGLLTGRPNSKATGP-----DGESLTAKEAFEKAGISTGFFDLQPKEG 256
PzaPAL      SASGDLSPLSYVAGALAGQRGIYCWVTDKKSGQRVKVTADEACRMHGIEPVL--YEPKEA 261
RtPAL       SASGDLSPLSYIAAAISGHPDSKVHVVHE--GKEKILYAREAMALFNLEPVV--LGPKEG 265
RgPAL       SASGDLSPLSYIAASITGHPDSKVHVD------GKIMSAQEAIALKGLQPVV--LGPKEG 267
KkPAL       GASGDLTPLSYVAAALIGER---EVLY-----KGQTQPTEQVFKSLGIKPIT--LQPKEG 197
PbPAL       GASGDLVPLGVIARSIIGHPSTTQVKY-----QGEQADSHDVLQQLNYSALQ--LEAKEG 204
AvPAL       GASGDLVPLSYITGSLIGLDPSFKVDF-----NGKEMDAPTALRQLNLSPLT--LLPKEG 218
AL-11       GASGDLVPLTYIGASILGLSPEFLVDL-----DGETLDCHAVLARLGFAPMD--PDPKEG 195
             ***** **  :   : *                       .    .          **.

PcPAL       LALVNGTAVGSGMASMVLFEANILAVLAEVMSAIFAEVMQGKPE-FTDHLTHKLKHHPGQ 314
AtPAL       LALVNGTAVGSGMASMVLFEANVQAVLAEVLSAIFAEVMSGKPE-FTDHLTHRLKHHPGQ 315
PzaPAL      LGLLNGTAFSASVAGLATYEAEKLAQLTQLTTAMAVEALKGTDASFAPFIHEVARPHPGQ 321
RtPAL       LGLVNGTAVSASMATLALHDAHMLSLLSQSLTAMTVEAMVGHAGSFHPFLHDVTRPHPTQ 325
RgPAL       LGLVNGTAVSASMATLALTDAHVLSLLAQALTALTVEAMVGHAGSFHPFLHDVTRPHPTQ 327
KkPAL       LAIMNGTAVMTALACLAFQRADYLTQLCSRITSLCSIALQGNSAHFDELLFS-VKPHPGQ 256
PbPAL       LALVNGTSFSSAIAANCVFESQRLLSLSLVLQSIMVRALGGHPEAFHPFVDE-NKPHPGQ 263
AvPAL       LAMMNGTSVMTGIAANCVYDTQILTAIAMGVHALDIQALNGTNQSFHPFIHN-SKPHPGQ 277
AL-11       LALNNGTGACTGVAANVMARALNAATMALGVHALFAQALLATDQSFDPYIHA-QKPHPGQ 254
            *.: ***.  :.:*      :     :     ::   .: .    *   :    : ** *

PcPAL       IEAAAIMEHILDGSAYVKAAQK---LHEMDPLQKPKQDRYALRTSPQWLGPQIEVIRSST 371
AtPAL       IEAAAIMEHILDGSSYMKLAQK---VHEMDPLQKPKQDRYALRTSPQWLGPQIEVIRQAT 372
PzaPAL      IKSARYIRALLSGSKLAEHLENEKHVLFSEDNGTLRQDRYTLRTASQWVGPGLEDIENAK 381
RtPAL       IEVAGNIRKLLEGSRFAVHHEEE--VKVKDDEGILRQDRYPLRTSPQWLGPLVSDLIHAH 383
RgPAL       IEVARNIRTLLEGSKYAVHHETE--VKVKDDEGILRQDRYPLRCSPQWLGPLVSDMIHAH 385
KkPAL       NQVAAWIRD----------DLN---HYKHPRNSDRLQDRYSIRCAPHIIGALKDAMPWMR 303
PbPAL       GWSAQMMRDLLSYSP-----------NDSKRNGDLAQDRYSLRCLAQYFAPIVEGIAQIS 312
AvPAL       LWAADQMISLLANSQLVRDELD---GKHDYRDHELIQDRYSLRCLPQYLGPIVDGISQIA 334
AL-11       VWSAARMADLLKDGRTIRSEAG---GDRARRKGDLIQDRYGIRCLPQFFGPIVDGLSTAA 311
               *  :                             **** :*   : ..   . :

PcPAL       KMIEREIN-SVNDNPLIDVS--RNKAIHGGNFQGTPIGVSMDNTRLAIAAIGKLMFAQFS 428
AtPAL       KSIEREIN-SVNDNPLIDVS--RNKAIHGGNFQGTPIGVSMDNTRLAIAAIGKLMFAQFS 429
PzaPAL      RSVDIEIN-STTDNPMIDPYDADGRIHHGGNFQAMAMTNAVEKIRLALCAMGKMTFQQMT 440
RtPAL       AVLTIEAGQSTTDNPLIDVE--NKTSHHGGNFQAAAVANTMEKTRLGLAQIGKLNFTQLT 441
RgPAL       AVLSLEAGQSTTDNPLIDLE--NKMTHHGGAFMASSVGNTMEKTRLAVALMGKVSFTQLT 443
KkPAL       QTIETELN-SANDNPIIDGA--GQHVLHGGHFYGGHIAMVMDSMKTGIANLADLMDRQMA 360
PbPAL       QSISTEMN-AVSDNPLIDVD--TGRFHQSGNFLGQYVAMSMDQLRRHLGLLAKHLDVQIA 369
AvPAL       KQIEIEIN-SVTDNPLIDVD--NQASYHGGNFLGQYVGMGMDHLRYYIGLLAKHLDVQIA 391
AL-11       RQIETEAN-TANDNPLINPE--TGETFHTGNFLAQYTAIAMDSTRYLIGLMCKHIDSQIA 368
              :  * . :..***:*:         : * * .      ::  :  :  : .    *::
```

```
PcPAL    ELVNDFYNNGLPSNLSGG--RNPSLDYGFKGAEIAMASYCSELQFLANPVTNHV-QSAEQ 485
AtPAL    ELVNDFYNNGLPSNLTAS--SNPSLDYGFKGAEIAMASYCSELQYLANPVTSHV-QSAEQ 486
PzaPAL   ELVNPAMNRGLPANLAST--PDLSLNFHAKGIDIALASVTSELMFLGNPVSTHV-QSAEM 497
RtPAL    EMLNAGMNRGLPSCLAA---EDPSLSYHCKGLDIAAAAYTSELGHLANPVTTHV-QPAEM 497
RgPAL    EMLNAGMNRALPSCLAA---EDPSLSYHCKGLDIAAAAYTSELGHLANPVSTHV-QPAEM 499
KkPAL    LLVDSKFNNGLPNNLSAASEQRRPLNHGFKAVQIGVSAWTAEALKLT-MPASVFSRSTEC 419
PbPAL    QLVAPAFNNGLPASLRGN--SSRPFNMGLKGLQITGNSIMPLLTYLGNPLTEHFPTHAEE 427
AvPAL    LLASPEFSNGLPPSLLGN--RERKVNMGLKGLQICGNSIMPLLTFYGNSIADRFPTHAEQ 449
AL-11    LMITPAFSNGLTPALVGN--METGVNVGLKSLHIGMNQMSTQISYLGQSVADRFPTHAEM 426
          :     ...*   * .       ..   *. .*                :  .   :*

PcPAL    HNQDVNSLGLISSRKTSEAVEILKLMSTTFLVGLCQAIDLRHLEENLKS-TVKNTVSSVA 544
AtPAL    HNQDVNSLGLISSRKTSEAVDILKLMSTTFLVGICQAVDLRHLEENLRQ-TVKNTVSQVA 545
PzaPAL   ANQAINSLALISGRQTLQAVECLSMIQAWSLYLLCQALDIRALQYKVAE-QLPAMVLASI 556
RtPAL    ANQAVNSLALISARRTTESNDVLSLLLATHLYCVLQAIDLRAIEFEFKK-QFGPAIVSLI 556
RgPAL    GNQAINSLALISARRTAEANDVLSLLLATHLYCVLQAVDLRAMEFEHTK-AFEPMVTELL 558
KkPAL    HNQDKVSMGTIAARDCLRILDLTEQVAAASLMAATQAVTLRIKQSQLDKSSLSDGVLSTL 479
PbPAL    FNQNINGLSWGSANLAWRSVQLFQHYLSVASIFAVQAIDLRAGLEADHC-DGRELLGETA 486
AvPAL    FNQNINSQGYTSATLARRSVDIFQNYVAIALMFGVQAVDLRTYKKTGHY-DARACLSPAT 508
AL-11    YNQNINSQAMNAANLARDQMDVTEHFLAAALLTAVQAVEVRSRVETGSC-DARDILSPAT 485
          **   . .  :.       :  .   :       **: :*              :
```

Figure S1. Section of a sequence alignment of PALs of different origins: *Pc*PAL (Uniprot code: P24481), *Av*PAL (Uniprot code: Q3M5Z3), *At*PAL2 (Uniprot code: P45724), *Pb*PAL (GenPept: WP_013629471), *Rt*PAL (Uniprot code: P11544), *Rg*PAL (Uniprot code: V5TFQ0), *Kk*PAL (Uniprot code: C7R9W9), *Pza*PAL (Uniprot code: M9M0D4) and AL-11 (GenBank: MW026687). The amino acid residues of the hydrophobic substrate binding region are shown in yellow background, while the grey background indicate the residues of the polar substrate binding region. The Ala-Ser-Gly sequence, responsible for the posttranslational production of the MIO prosthetic group is marked with pink background. The amino acids from *Pc*PAL, *Rt*PAL and *At*PAL subjected to mutations within this study are marked with blue (for *ortho* substituents), orange (for *meta* substituents), green (for *para* substituents) colours, while differences related to these residues in PALs of different origins are marked with red colour.

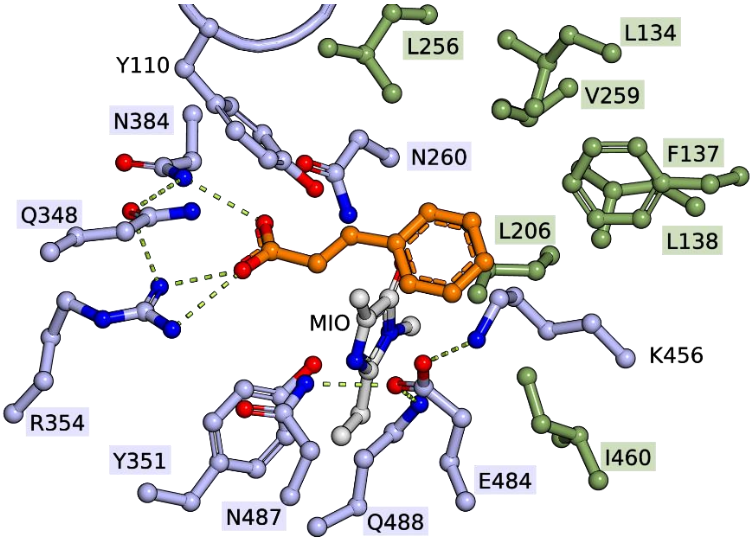

Figure S2. Catalytic site of *Pc*PAL (PDB ID: 6F6T), with cinnamic acid as ligand. Key residues from the hydrophobic substrate binding region, accommodating the aromatic moiety of substrates, are marked in green. Residues of the polar substrate binding region, accommodating the MIO-prosthetic group is stabilized through several H-bonds, being involved in fixation of the -COOH, and $-NH_2$ (in case of Phe substrate analogues) functionalities

4. Site-directed mutagenesis

The site-directed mutagenesis was performed following the protocol described by Naismith and Liu.[3] The *Pc*PAL mutants were obtained as described in our previous work[4] while the *At*PAL and *Rt*PAL mutants were optimized accordingly. The PCR reaction contained 2 ng of DNA template (recombinant plasmid encoding *Rt*PAL or *At*PAL), 1 μM solution of primer pair (**Table S1**), 200 μM dNTPs and 0.5 μL of DNA Phusion Hot Start polymerase, filled up to 50 μL with water. The PCR cycles were initiated at 95 °C for 3 min, followed by 25 amplification cycles. Each amplification cycle consisted of 95 °C for 1 min, at temperature of T_m^{no}-5 °C for 1 min and 72 °C for 8 min. The PCR cycles were finished with an annealing step at T_m^{pp}-5 °C for 1 min and the final extension step at 72 °C for 15 min. 10 μL of each PCR reactions was analyzed by agarose gel electrophoresis, followed by treatment with 5 units of *Dpn*I at 37 °C for 1 h 30 min. An aliquot of 3 μL from the above PCR product was transformed into 100 μL suspension of *E. coli* XL1-Blue competent cells by heat shock. The transformed cells were spread on a Luria-Bertani (LB) plate containing carbenicillin (50 μg/mL) and tetracycline (12.5 μg/mL) and incubated at 37 °C overnight. Two colonies from each plate were grown in 5 mL sterile LB medium containing the corresponding antibiotics at 37 °C overnight and the DNA plasmid was isolated using GenElute Plasmid Miniprep Kit from Sigma Aldrich. DNA sequencing was carried out using the Sequencing Service of Biomi (Gödöllő, Hungary).

Table S1. List of the obtained *At*PAL and *Rt*PAL mutants and the primers used for mutagenesis.

Entry	Mutant	Sequence (5'-3') of mutagenic primers	Template DNA
**1**	***Rt*PAL_L134A**	*RtPAL_L134A-for:* GAAGGCCGCGTTGGAACATCAGTTGTGCGGAGTATTGCCCTCC	*wt-rtpal*
		RtPAL_L134A-rev: GTTCCAACGCGGCCTTCTGCAAGGAAATGGCGTCTTCAGTTCTAGTG	
**2**	***Rt*PAL_H137V**	*RtPAL_H137V-for:* GTTGGAAGTTCAGTTGTGCGGAGTATTGCCCTC	*wt-rtpal*
		RtPAL_H137V-rev: ACAACTGAACTTCCAACAAGGCCTTCTGCAAGG	
**3**	***Rt*PAL_L266V**	*RtPAL_L266V-for:* GAAGGAGTGGGATTGGTAAACGGAACTGCCGT	*wt-rtpal*
		RtPAL_L266V-rev: CAATCCCACTCCTTCCTTGGGTCCCAATACTACG	
**4**	**RtPAL_I472V**	*RtPAL_I472V-for:* TGGACGTTGCCGCCGCCGCCTACACTTCCG	*wt-rtpal*
		RtPAL_I472V-rev: GGCGTTCAAGACTCTAATCAATTCCTTCTGCAAGGCTCC	
**5**	***Rt*PAL_H137F/Q138L**	*RtPAL_H137F/Q138L-for:* TGGAATTTCTGTTGTGCGGAGTATTGCCCTCC	*wt-rtpal*
		RtPAL_H137F/Q138L-rev: CACAACAGAAATTCCAACAAGGCCTTCTGCAAG	
**6**	***Rt*PAL_H137V/Q138L**	*RtPAL_H137V/Q138L-for:* TGGAAGTTCTGTTGTGCGGAGTATTGCCCTCC	*wt-rtpal*
		RtPAL_H137V/Q138L-rev: CACAACAGAACTTCCAACAAGGCCTTCTGCAAG	
**7**	***Rt*PAL_I472V/H137F/Q138L**	*RtPAL_I472V-for:* TGGACGTTGCCGCCGCCGCCTACACTTCCG	*H137F/Q138L*
		RtPAL_I472V-rev: GGCGTTCAAGACTCTAATCAATTCCTTCTGCAAGGCTCC	
**8**	***Rt*PAL_L266V/H137F/Q138L**	*RtPAL_L266V-for:* GAAGGAGTGGGATTGGTAAACGGAACTGCCGT	*H137F/Q138L*
		RtPAL_L266V-rev: CAATCCCACTCCTTCCTTGGGTCCCAATACTACG	

**9**	***At*PAL_L133A**	*AtPAL_L133A-for:* ACAGAAGCCATTAGATTTTTGAACGCCGGAATATTCGG	*wt-atpal*
		AtPAL_L133A-rev: CAAAAATCTAATGGCTTCTGTTTGTAATGCGGTGCCG	
**10**	***At*PAL_L257V**	*AtPAL_L257V-for:* AGGAAGGTGTAGCTCTCGTTAATGGCACGGCG	*wt-atpal*
		AtPAL_L257V-rev: GAGAGCTACACCTTCCTTAGGTTGTAAATCGAAGAATCCA	
**11**	***At*PAL_F136V**	*AtPAL_F136V-for:* CTCATTAGAGTTTTGAACGCCGGAATATTCGGAAACAC	*wt-atpal*
		AtPAL_F136V-rev: CGTTCAAAACTCTAATGAGTTCTGTTTGTAATGCGGTG	
**12**	***At*PAL_I461V**	*AtPAL_I461V-for:* AGCAGAGGTTGCTATGGCTTCTTATTGTTCTGAGCTTC	*wt-atpal*
		AtPAL_I461V-rev: CATAGCAACCTCTGCTCCTTTGAATCCATAATCCA	

5. Protein expression, isolation and folding characterization

5.1. Expression and purification of PAL variants

Expression, isolation and purification of all PAL proteins was performed according to our optimized protocol.[5] The His-tagged proteins were eluted from the affinity chromatography resin (Ni-NTA Agarose, Qiagen) with the 250 mM imidazole solution as elution buffer (made in LS- low salt buffer: 50 mM HEPES, 30 mM KCl, pH 7.5). After dialysis (20 mM Tris, 100 mM NaCl, pH 7.5), desalting and protein concentration step, protein samples were analysed by SDS-PAGE, while protein concentrations were assessed by the Bradford method.

5.2. Thermal unfolding profile of PALs

Table S2. Melting temperatures (T_m) of *Rt*PAL and *At*PAL variants compared with the reported values for *Pc*PAL analogues[4].

**Nr. Crt.**	***Pc*PAL**	T_m (°C)	***At*PAL**	T_m (°C)	***Rt*PAL**	T_m (°C)
1	*wild-type*	75.1±0.2	*wild-type*	73±0.1	*wild-type*	70.7±0.1
2	L134A	70.2±0.5	L133A	71.5±0.5	L134A	70.8±0.4
3	F137V	73.5±0.4	F136V	71.1±1.0	H137V	72.7±0.2
4	L256V	73.1±0.5	L257V	72.1±0.6	L266V	71.8±1.0
5	I460V	74.2±0.2	I461V	72.5±1.0	I472V	69.4±0.2
6					H137F/Q138L/L266V	70.8±0.2
7					H137F/Q138L/I472V	65.0±2.0
8					H137F/Q138L	71.5±0.2
9					H137V/Q138L	71.2±0.2

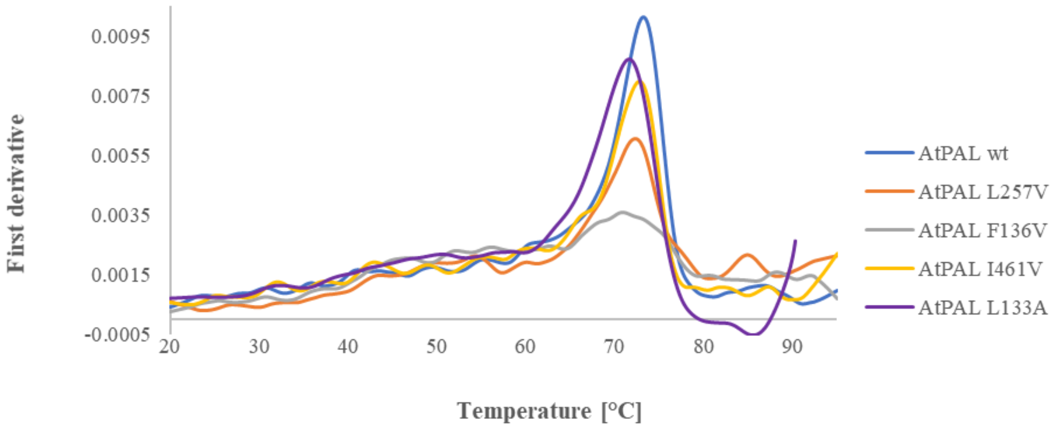

Figure S3. Thermal unfolding of *wild-type* and *At*PAL variants L133A, L257V, F136V and I461V. Melting temperatures (T_m) range from 71 to 73 ºC.

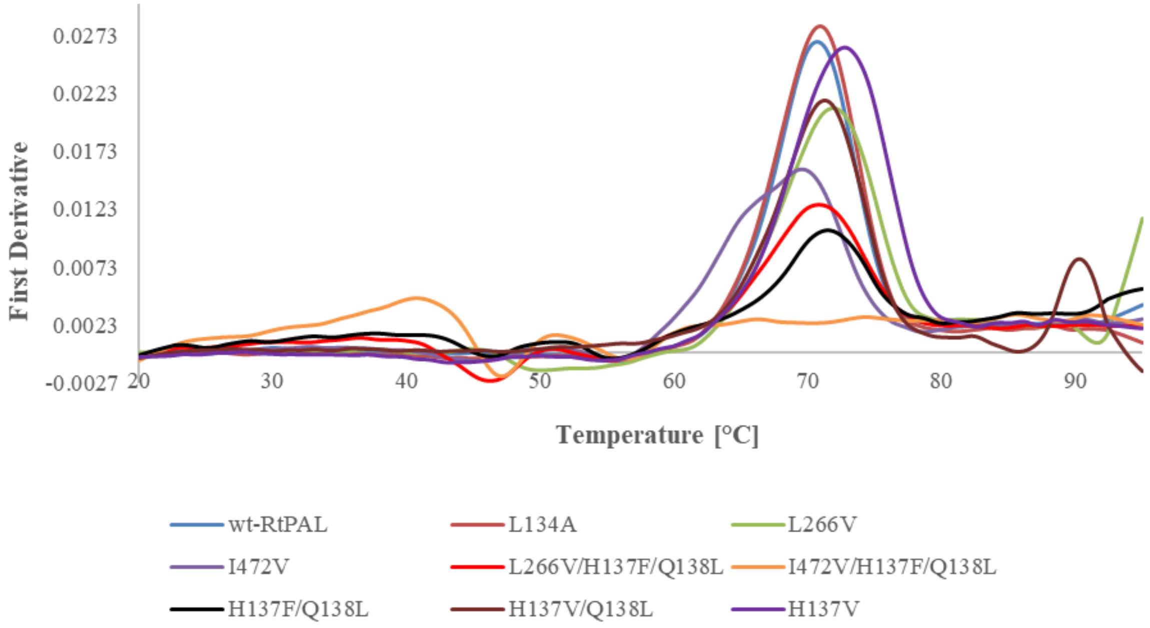

Figure S4. Thermal unfolding of *wild-type* and *Rt*PAL variants H137V, L134A, I472V, L266V, H137F/Q138L, H137V/Q138L, H137F/Q138L/L266V and H137F/Q138L/I472V. Melting temperatures (T_m) range from 65 to 72 ºC.

6. HPLC monitoring of the enzymatic reactions

All analytical scale biotransformations were performed in duplicates, while during the initial activity screens using a significantly sized reaction-subset the HPLC analysis have been performed for all samples within the duplicate set (**Table S3**). All other HPLC analysis were performed only for a single sample set taken from the duplicate reactions, but in each measurement set some randomly chosen duplicates were also included, in order to support the data values obtained.

Table S3. The conversions of the PAL-catalyzed ammonia addition reactions (duplicate values) of substrates **2a**, **2e**, **2i**.

Subst.	Enzyme	Variant	Conversion (%) S1*	Conversion (%) S2*	Reaction time (h)
***o*-Br 2a**	***Pc*PAL**	***wt***	94.1	97.3	24
		**L256V**	94.6	96.8	24
	***At*PAL**	***wt***	93.9	94.7	1
		**L257V**	94.6	90.8	1
	***Rt*PAL**	***wt***	30.7	28.6	6
		**L266V**	94.2	96.4	6
		**H137F/Q138L/ L266V**	96.3	92.3	6
***m*-Br 2e**	***Pc*PAL**	***wt***	84.8	84.0	24
		**I460V**	89.6	92.0	24
		**L134A**	88.6	90.6	24
	***At*PAL**	***wt***	80.2	75.7	3
		**I461V**	89.0	85.7	3
		**L133A**	87.0	88.1	3
	***Rt*PAL**	***wt***	86.4	81.1	6
		**I472V**	36.8	41.4	24
		**L134A**	91.9	84.5	6
		**H137F/Q138L/I472V**	<1	<1	6
***p*-Br 2i**	***Pc*PAL**	***wt***	7.6	6.6	24
		**I460V**	57.5	60.0	24
		**F137V**	71.4	71.8	24
	***At*PAL**	***wt***	79.8	89.6	16
		**I461V**	90.6	90.4	16
		**F136V**	88.6	90.2	16
	***Rt*PAL**	***wt***	<1	<1	16
		**I472V**	<1	<1	16
		**H137V**	14.8	22.2	16
		**H137F/Q138L/I472V**	<1	<1	16
		**H137V/Q138L**	85.5	83.1	16

*S1-sample 1, S2- sample 2 (duplicate)

7. Enzyme kinetic measurements – standard deviations

The initial enzyme activities were spectrophotometrically determined, using a Tecan Infinite Spark 10 M microplate reader and Corning 96-well Clear Flat Bottom UV-Transparent microplates. The kinetic measurements were performed in triplicate at 30 °C by monitoring the production of *trans*-cinnamic acid analogues **2a-l** at 290 nm (wavelength where the corresponding amino acids ***rac*-1a-l** showed no absorption), using substrate concentrations of 0.1-20 mM of **1a-l**, 100 mM Tris.HCl, 120 mM NaCl (pH 8.8) as buffer and purified PAL variants at fixed enzyme concentration of 0.322 μM. Kinetic constants (K_M, v_{max}) were obtained from the Michaelis-Menten curves by non-linear fitting using GraphPad Prism. Standard deviation for the calculated K_M and k_{cat} values are given in **Tables S4-S6.**

Table S4. Standard deviations for the enzyme kinetic obtained for the ammonia elimination reactions of *ortho*-substituted *rac*-phenylalanines **1a-d**.

Subst.	Enzyme	Variant	K_M (μM)	k_{cat} (s^{-1})
***o*-Br 1a**	***Pc*PAL**	***wt***	153±37	0.157±0.011
		**L256V**	110±7.3	0.365±0.003
	***At*PAL**	***wt***	199±7	0.21±0.006
		**L257V**	184±18	0.435±0.006
	***Rt*PAL**	***wt***	662±62	0.094±0.002
		**L266V**	254±8	0.079±0.001
		**H137F/Q138L/L266V**	59±8.5	0.046±0.003
***o*-CF_3 1b**	***Pc*PAL**	***wt***	523±5.3	0.042±0.0002
		**L256V**	2733±339	0.148±0.011
	***At*PAL**	***wt***	240±36	0.032±0.0001
		**L257V**	2911±137	0.177±0.052
	***Rt*PAL**	***wt***	92±10.1	0.004±0.002
		**L266V**	1225±85	0.019±0.002
		**H137F/Q138L/L266V**	103±4	0.117±0.004
***o*-OCH_3 1c**	***Pc*PAL**	***wt***	n.d.	n.d.
		**L134A**	1254±86	0.108±0.003
	***At*PAL**	***wt***	4752±251	0.019±0.005
		**L133A**	326±21	0.058±0.003
	***Rt*PAL**	***wt***	2580±236	0.014±0.005
		**L134A**	6552±656	0.016±0.009
		**H137F/Q138L/L266V**	n.d.	n.d.
***o*-CH_3 1d**	***Pc*PAL**	***wt***	59±12	0.211±0.001
		**L256V**	128±20	0.282±0.007
	***At*PAL**	***wt***	46±14.4	0.119±0.003
		**L257V**	n.d.	n.d
	***Rt*PAL**	***wt***	663±29	0.252±0.004
		**L266V**	760±65	0.268±0.007
		**H137F/Q138L/L266V**	96±5.4	0.115±0.005

n.d. – not determinable, during enzyme kinetics the non-linear range of the Michaelis-Menten curve was not obtained using substrate concentration allowed by the solubility of the tested compounds

Table S5. Standard deviations for the enzyme kinetic obtained for the ammonia elimination reactions of *meta*-substituted *rac*-phenylalanines **1e-h**.

Subst.	Enzyme	Variant	K_M (µM)	k_{cat} (s^{-1})
***m*-Br 1e**	***Pc*PAL**	***wt***	153±4.3	0.095±0.004
		**I460V**	51±9.2	0.154±0.005
		**L134A**	-	-
	***At*PAL**	***wt***	n.d.	n.d.
		**I461V**	55±15	0.197±0.009
		**L133A**	76±7	0.395±0.0007
	***Rt*PAL**	***wt***	363±25	0.343±0.006
		**I472V**	n.d.	n.d.
		**L134A**	409±36	0.288±0.006
		**H137F/Q138L/I472V**	n.d.	n.d.
***m*-CF_3 1f**	***Pc*PAL**	***wt***	533±24	0.057±0.002
		**I460V**	163±29	0.204±0.011
		**L134A**	912±6.4	0.203±0.011
	***At*PAL**	***wt***	217±31	0.04±0.001
		**I461V**	112±20	0.272±0.004
		**L133A**	-	-
	***Rt*PAL**	***wt***	1369±176	0.11±0.005
		**I472V**	2505±104	0.013±0.0004
		**L134A**	1573±74	0.085±0.001
		**H137F/Q138L/I472V**	-	-
***m*-OCH_3 1g**	***Pc*PAL**	***wt***	378±21	0.011±0.027
		**L134A**	170±31	0.431±0.035
	***At*PAL**	***wt***	297±28	0.095±0.006
		**L133A**	49±11	0.213±0.003
	***Rt*PAL**	***wt***	2971±261	0.078±0.007
		**L134A**	4291±325	0.077±0.006
***m*-CH_3 1h**	***Pc*PAL**	***wt***	55±21	0.014±0.0009
		**L134A**	115±18	0.100±0.007
	***At*PAL**	***wt***	n.a.	n.a.
		**L133A**	63±11	0.113±0.001
	***Rt*PAL**	***wt***	333±19	0.153±0.007
		**L134A**	338±21	0.146±0.011

n.d. – not determinable, during enzyme kinetics the non-linear range of the Michaelis-Menten curve was not obtained using substrate concentration allowed by the solubility of the tested compounds
n.a. – no activity detected
"-" – no determination/measurement was performed

Table S6. Standard deviations for the enzyme kinetic obtained for the ammonia elimination reactions of *para*-substituted *rac*-phenylalanines **1i-l**.

Subst.	Enzyme	Variant	K_M (µM)	k_{cat} (s^{-1})
***p*-Br 1i**	***Pc*PAL**	***wt***	269±20	0.165±0.002
		**I460V**	71±5.1	0.259±0.011
		**F137V**	-	-
	***At*PAL**	***wt***	73±2.1	0.085±0.004
		**I461V**	61±2.5	0.245±0.017
		**F136V**	-	-
	***Rt*PAL**	***wt***	435±8.2	0.005±0.0002
		**I472V**	2120±74	0.008±0.009
		**H137V**	-	-
		**H137F/Q138L/I472V**	n.a.	n.a.
		**H137V/Q138L**	n.a.	n.a.
***p*-CF_3 1j**	***Pc*PAL**	***wt***	2490±87	0.25±0.003
		**I460V**	901±64	0.55±0.033
		**F137V**	151±2.5	0.42±0.007
	***At*PAL**	***wt***	1467±123	0.127±0.014
		**I461V**	275±54	0.428±0.02
		**F136V**	-	-
	***Rt*PAL**	***wt***	6381±255	0.004±0.0001
		**I472V**	n.a.	n.a.
		**H137V**	3231±132	0.032±0.0003
		**H137F/Q138L/I472V**	n.a.	n.a.
		**H137V/Q138L**	n.a.	n.a.
***p*-OCH_3 1k**	***Pc*PAL**	***wt***	1858±95	0.009±0.002
		**I460V**	265±24	0.103±0.004
		**F137V**	-	-
	***At*PAL**	***wt***	1048±27	0.007±0.0001
		**I461V**	132±19	0.112±0.007
		**F136V**	-	-
	***Rt*PAL**	***wt***	n.d.	n.d.
		**I472V**	n.d.	n.d.
		**H137V**	n.d.	n.d.
		**H137F/Q138L/I472V**	-	-
		**H137V/Q138L**	-	-
***p*-CH_3 1l**	***Pc*PAL**	***wt***	208±37	0.026±0.0004
		**I460V**	107±20	0.092±0.004
		**F137V**	-	-
	***At*PAL**	***wt***	191±25	0.018±0.0007
		**I461V**	144±15	0.1±0.003
		**F136V**	-	-
	***Rt*PAL**	***wt***	9902±550	0.003±0.0002
		**I472V**	n.a.	n.a.
		**H137V**	7080±123	0.003±0.00008
		**H137F/Q138L/I472V**	n.a.	n.a.
		**H137V/Q138L**	n.a.	n.a.

n.d. – not determinable, during enzyme kinetics the non-linear range of the Michaelis-Menten curve was not obtained using substrate concentration allowed by the solubility of the tested compounds
n.a. – no activity detected
"-" – no determination/measurement was performed

8. Computational studies

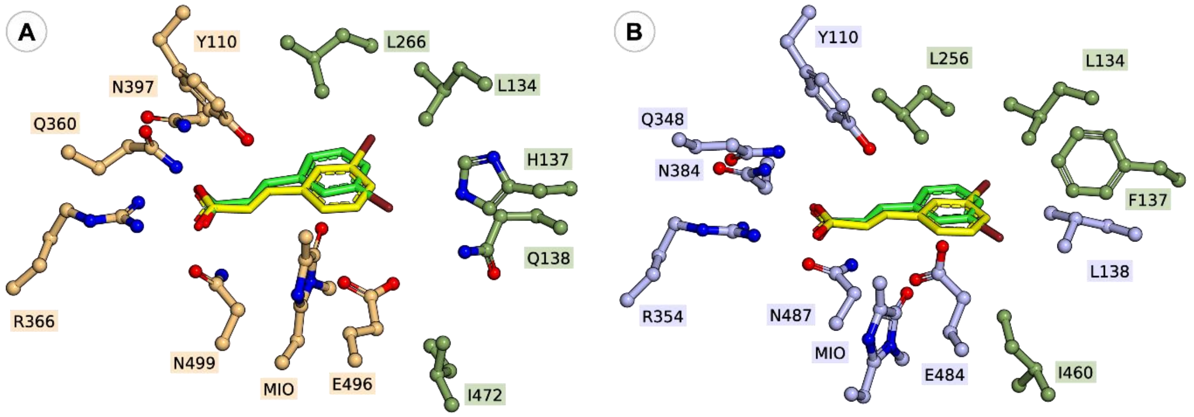

Figure S5. The lowest energy active orientations of *m*-Br-cinnamic acid **2e** within catalytic site of **(A)** *wt-Rt*PAL and **(B)** *wt-Pc*PAL, exposing the *m*-Br-substituent towards residues L134 (yellow orientation of **2e**) or I472/I460 (green orientation of **2e**) of *Rt*/*Pc*PAL.

Table S7. Binding energies of the active substrate orientations (ASO) of *meta*-substituted cinnamic acids **2e**-**2h**, exposing the *meta*- substituent towards residues I472/I460 or L134 within the active site of *wild-type Rt*PAL and *Pc*PAL.

Substrate	***wt-Rt*PAL**		***wt-Pc*PAL**	
	ASO_{I472} (*kcal/mol*)	ASO_{L134} (*kcal/mol*)	ASO_{I460} (*kcal/mol*)	ASO_{L134} (*kcal/mol*)
**2e**	-5.5	-4.8	-6.9	-6.4
**2f**	-6.3	-5.4	-8.5	-6.4
**2g**	-5.1	-4.9	-6.9	n.o.*
**2h**	-6.0	-6.8	-8.0	-7.8

n.o.*- no active substrate orientation with the aromatic substituent pointing towards the specific residue has been obtained

9. References

1. Nagy, E. Z. A. *et. al.* Mapping the hydrophobic substrate binding site of phenylalanine ammonia lyase from *Petroselinum crispum*, *ACS Catal.* **9**, 8825-8834 (2019).
2. Madeira, F. *et al.* The EMBL-EBI search and sequence analysis tools APIs in 2019. *Nucleic Acids Res.* **47**, W636-W641 (2019).
3. Liu, H. & Naismith, J. H. An efficient one-step site-directed deletion, insertion, single and multiple-site plasmid mutagenesis protocol, *BMC Biotechnol.* **8**, 91–101 (2008).
4. Filip, A. *et. al.* Tailored mutants of phenylalanine ammonia-lyase from *Petroselinum crispum* for the synthesis of bulky L- and D-arylalanines, *ChemCatChem.* **10**, 2627-2633 (2018).
